# Supplementary material for: Understanding and Designing Phase Change Materials: Insights From Atom Probe Tomography
Source: Adv Sci (Weinh). 2026 Sep 30:e00082. Online ahead of print. doi: 10.1002/advs.202600082 (PMC13624867; doi:10.1002/advs.202600082)
Supplement: Supplementary file 1 [file ADVS-9999-e00082-s001.docx]

# **Supporting Information**

# **Understanding and Designing Phase Change Materials: Insights from Atom Probe Tomography**

#### Jan Köttgen^1^, Nils von den Driesch^2^, Alexander Pawlis^2^, Matthias Wuttig^1,2^

**J. Köttgen, Prof. M. Wuttig**

^1^ Institute of Physics IA, RWTH Aachen University, 52074 Aachen, Germany

* E-Mail: wuttig@physik.rwth-aachen.de

**Dr. N. von den Driesch, Dr. A. Pawlis, Prof. M. Wuttig**

^2^ Peter-Grünberg-Institute – JARA-Institute Energy Eﬃcient Information Technology (PGI-10) Wilhelm-Johnen-Straße, 52428 Jülich, Germany

## Local Electrode Atom Probe Tomography

The Local Electrode Atom Probe (LEAP) comprises a local electrode, a pulsing near-UV laser, and a position-sensitive detector. The specimen's tip, with a diameter below 100 nm, is cooled to cryogenic temperatures (typically 30-60K) within an ultra-high vacuum chamber. Positioned approximately 10 µm from the tip-shaped specimen, the local electrode applies a static voltage in the range of a few thousand volts. To compensate for the increasing tip diameter with ongoing evaporation, the voltage rises continuously to apply a constant electric field. A voltage or laser pulse initiates the evaporation at specific time intervals; ions are then emitted and accelerated from the specimen surface towards the position-sensitive detector. The ions' time-of-flight between pulse initiation and detector impact correlates with their mass to charge ratio, facilitating the determination of chemical identities.

## Evaporation Descriptors

The field evaporation of different compounds can be described by different quantities. The Probability of Multiple Events (PME) is described as the ratio of the number of ions that are detected on the same pulse as another ion ($X_{ME}$) to the total number of detected ions consisting of detected ions from multiple events $X_{ME}$ and single events $X_{SE}.$ On the latter, only one ion is detected during an evaporation event. Probability of Molecular Ions (PMI) is described as the ratio of detected molecular ions ($X_{MI}$) to the total number of detected ions consisting of the number of molecular ions $X_{MI}$ and single ions $X_{SI}$, consisting of only one atom.

## Mean Fragment Size

In addition to the two previously introduced bond rupture descriptors (PME and PMI), the mean fragment size (MFS) is used to further investigate the evaporation behavior. It is defined as the arithmetic mean of the number of atoms per fragment $a_{i}$ in regards to the total number of fragments $n_{i}$ Figure S1 gives an example for the analysis of the relevant quantities.

## Determination of the PME

The goal for the PME determination is an experiment with the lowest amount of uncorrelated evaporation and molecular dissociation. Therefore, each material is measured using an individual set of parameters, with a focus on optimizing the laser pulse energy. The laser pulse frequency used is typically rather low, as some of the metavalent and covalent materials evaporate with very large molecular ions, resulting in a high time-of-flight. Each experiment is analyzed by investigating the corresponding correlation histogram. If DC-evaporation (trails starting from the origin) are observed, the laser pulse energy is raised to increase the number of ions that evaporate immediately after the laser pulse impact. If the tails of the mass peaks are elongated, indicating a high tip surface temperature induced by a high laser pulse energy, the latter is reduced. Thus, the highest number of ions evaporates directly after the laser pulse reaches the tip. Through this approach the materials can be characterized with the required attention due to their very unique evaporation behaviors and allows the determination of a distinct set of PME, PMI and MFS values.

In the case shown in Figure S5 the PME is extracted from the experiment performed at 10 pJ, as this the lowest laser pulse energy that shows no DV-evaporation.

## LEAP comparison

*The used laser wavelengths of the different LEAP setups were reduced with ongoing development of the devices. Lee et al. showed a difference of the PME measured via the Invizo 6000 compared with the LEAP 5000. However, due to the very different setup designs, this difference can not only be attributed to the different laser wavelengths. The differently shaped local electrodes combined with the Invizo’s dual laser setup and decelerating lenses make the influence of the wavelength hard to separate.*

*A more realistic comparison of the LEAP 5000 XR and the 6000 XR by Schiester et al. makes it obvious that the multiplicity histograms generated from the LEAP 5000 XR and 6000 XR are very similar.*

*Despite this evidence, we investigated the imaginary part of the dielectric function of the typical metavalent material GeTe as a function of the wavelength, shown in Figure S6: It shows that for higher wavelengths the imaginary part eps2 as a metric of the absorption increases. Thus, the laser energy can be absorbed even more for higher wavelengths, making the LEAP 3000, 4000 and 5000 the most suitable for the investigation of metavalent materials.*

*We can also consider another aspect of the varying available LEAP setups:*

*The detectors of the newest LEAP generation (LEAP 5000 & 6000) have an open detector area of 80%. When using a reflectron (LEAP 6000 XR and LEAP 5000 XR), the resulting efficiency drops to 52%. The previous generation of detectors (LEAP 3000 & 4000) had an open area of 55%, which is reduced to 37% with the use of a reflectron.*

*All data is gathered with a LEAP 5000 XS, meaning that the detector efficiency is as high as possible and thus causing the lowest uncertainty that a multiple event is actually observed as a multiple event. We will discuss this point in more detail in the answer of question #6.*

*Compare with* [*https://www.sciencedirect.com/science/article/pii/S030439912500004X*](https://www.sciencedirect.com/science/article/pii/S030439912500004X)

## PME uncertainty determination

Every evaporated ion is observed with a probability of ε on the detector, given by the open area of the detector and the time flight (ε = 80% for a LEAP 5000 XS).

Considering an example of a double multi-hit evaporation, this event is detected as a multiple event with a probability of $\varepsilon^{2}$, as a single event with $2(1-\varepsilon)\varepsilon$ and as no event with ${(1-\varepsilon)}^{2}$. For the case of a LEAP 5000 XS this corresponds to 64% for the detection of a double event, 32% of a single event and 4% for the detection of no event.

The probability of a multiple event of the Multiplicity M being detected as a single event (k=1) can be generalized with the binomial coefficient as

$$P\left( k=1 | M \right)=\binom{M}{k} \varepsilon^{k}{(1-\varepsilon)}^{M-k}$$

For a given dataset with $\binom{M}{1}=M$ and the number of events i per Multiplicity $i_{n}$ we receive:

$$\Delta_{Multiples to Singles}=\sum_{n=1}^{M} n \varepsilon^{1}{(1-\varepsilon)}^{n-1} i_{n}$$

Analogously, we receive

$$\Delta_{Multiples to Zero}=\sum_{n=1}^{M} {(1-\varepsilon)}^{n} i_{n}$$

and

$$\Delta_{Singles to Zero}=(1-\varepsilon)i_{1}$$

We can then describe the uncertainty on the multiple events as

$\Delta_{ME}=\Delta_{Multiples to Singles}+\Delta_{Multiples to Zero}$

and the uncertainty on the single events as

$$\Delta_{SE}={- \Delta}_{Multiples to Singles}+\Delta_{Singles to Zero}$$

For the final uncertainty of the PME we propagate

$$PME=\frac{X_{ME}}{X_{ME}+ X_{SE}}$$

to

$$\sigma_{PME}= \frac{\sqrt{{{(X}_{SE} \Delta_{ME})}^{2}+{{(X}_{ME} \Delta_{SE})}^{2}}}{{{(X}_{SE}{+X}_{ME})}^{2}}$$

The corresponding uncertainties for a variety of values are displayed in Table S1. A statistical analysis is performed via a boxplot analysis in Figure S7.

**Figure S1:** Schematic depiction of the used atom probe tomography analysis. PME, PMI and MFS are defined and explained for an example measurement.

**Figure S2**: Dimensionless product *k*_F_ *⋅ λ* versus DC conductivity for a wide range of materials. The parameter *k*_F_ *⋅ λ* compares the electron mean free path (*λ*) to the Fermi wavelength (1/*k*_F_), serving as an indicator of metallic transport. Solid lines around *k*_F_ ⋅ *λ* = 1 mark the Mott-Ioffe-Regel limit, where metallic conduction gives way to localization as conductivity decreases. These data were used to define the gray bars in all main text figures.

**Figure S3:** Fully labeled version of Figure 2 in the main text. Characteristic pattern of bond rupture for different crystalline solids.

**Figure S4**: Fully labeled version of Figure 8 in the main text. Bond rupture (PME) for crystalline solids as a function of the electrical conductivity at room temperature. The typical uncertainty is displayed.

Figure S5: The PME is determined by using an experiment with no DC-evaporation or dissociation in the corresponding correlation histogram.

Figure S6: The imaginary part of the dielectric function of the metavalent compound GeTe shown with the different laser wavelengths of the commercial LEAPs.

| **Sample** | **Error PME (%)** | **PME (%)** |
| --- | --- | --- |
| AgBiSe2 | 3,7 | 58,3 |
| AgSnSe2 | 3,0 | 53,5 |
| AgSnTe2 | 2,5 | 69,8 |
| Al | 1,2 | 3,6 |
| As2Te3 | 1,9 | 75,0 |
| Bi2S3 | 1,1 | 3,4 |
| Bi2Se3 | 2,7 | 6,4 |
| Bi2Te3 | 1,2 | 8,1 |
| Bi | 6,4 | 59,0 |
| GST | 6,8 | 86,6 |
| GaSb | 2,8 | 10,4 |
| GaSe | 2,0 | 6,8 |
| GeSe | 4,2 | 19,8 |
| GeTe | 4,3 | 65,7 |
| IST | 9,6 | 84,4 |
| In2Se3 | 2,1 | 7,3 |
| InSb | 2,5 | 9,8 |
| InTe | 3,9 | 12,2 |
| PbO | 2,6 | 9,2 |
| PbS | 0,4 | 74,4 |
| PbSe | 0,5 | 70,8 |
| PbTe | 6,7 | 56,5 |
| Sb2S3 | 3,3 | 11,9 |
| Sb2Te3 | 3,0 | 67,3 |
| Sb | 1,3 | 79,0 |
| Sb | 0,2 | 75,9 |
| Si | 3,9 | 12,2 |
| SnSe | 4,4 | 18,8 |
| SnTe | 4,1 | 71,4 |
| Sn | 5,8 | 3,0 |

Table S1: The uncertainties for the PME of different solids.
